# Multiple regulation pathways and pivotal biological functions of STAT3 in cancer

**DOI:** 10.1038/srep17663

**Published:** 2015-12-03

**Authors:** Jie Yuan, Fei Zhang, Ruifang Niu

**Affiliations:** 1 Public Laboratory, Key Laboratory of Breast Cancer Prevention and Therapy, Ministry of Education, Tianjin Medical University Cancer Institute and Hospital, National Clinical Research Center for Cancer, Tianjin Medical University, Huan-Hu-Xi Road, Ti-Yuan-Bei, He Xi District, Tianjin, 300060, People’s Republic of China

## Abstract

STAT3 is both a transcription activator and an oncogene that is tightly regulated under normal physiological conditions. However, abundant evidence indicates that STAT3 is persistently activated in several cancers, with a crucial position in tumor onset and progression. In addition to its traditional role in cancer cell proliferation, invasion, and migration, STAT3 also promotes cancer through altering gene expression via epigenetic modification, inducing epithelial–mesenchymal transition (EMT) phenotypes in cancer cells, regulating the tumor microenvironment, and promoting cancer stem cells (CSCs) self-renewal and differentiation. STAT3 is regulated not only by the canonical cytokines and growth factors, but also by the G-protein-coupled receptors, cadherin engagement, Toll-like receptors (TLRs), and microRNA (miRNA). Despite the presence of diverse regulators and pivotal biological functions in cancer, no effective therapeutic inventions are available for inhibiting STAT3 and acquiring potent antitumor effects in the clinic. An improved understanding of the complex roles of STAT3 in cancer is required to achieve optimal therapeutic effects.

Cancer progression is a multistep and complex process that begins with abnormal cells with malignant potential or neoplastic characteristics and continues with tumor growth, stromal invasion, and metastasis. This event not only relies on tumor-intrinsic effects, but also the tumor microenvironment which includes surrounding and supportive stroma, humoral factors, different effectors of immune system, and vasculature. As a transcription activator and an oncogene, STAT3 which is frequently detected with persistent activation in most human cancer cell lines and tumor tissues, is crucial in tumor cell proliferation, invasion, and migration, and is capable of inducing epithelial–mesenchymal transition (EMT), regulating the tumor microenvironment and promoting CSCs self-renewal and differentiation which all benefit the progression of cancer. Recent studies illustrated that STAT3 can also regulate gene expression through epigenetic modification, such as regulating the chromatin organization by unphosphorylated STAT3^1^ and contributing to the silencing of tumor-suppressor genes via DNA methylation by acetylated STAT3^2^,^3^. Much evidence has revealed the central cancer-promoting role of STAT3, thereby making it an ideal target for cancer therapy. However, despite the numerous regulators and pivotal biological functions in cancer, effective therapeutic inventions to inhibit STAT3 and to achieve potent antitumor effects in the clinic have not been identified and still need to be explored further. Therefore, a comprehensive exploration of the complicated biological behaviors of STAT3 in cancer is direly needed to inhibit the STAT3 signaling pathway.

## Novel insights into the regulation of STAT3

### Cytokine receptors, receptor tyrosine kinases, and non-receptor tyrosine kinases

Cytokine receptors which function as receptors for the interleukin-6 (IL-6) family cytokines are the most well-known traditional activators of STAT3. The interleukin-6 (IL-6) family cytokines function as ligands bind to a corresponding receptor to induce the homodimerization or the heterodimerization of gp130. After dimerization of the gp130 receptor complex, Janus kinases (JAK) are catalytically activated and transphosphorylate tyrosine residues in the gp130 receptor intracellular domain. Subsequently, the gp130 receptor complex recruits STAT3 to docks to the phosphorylated residues of the receptor via the SH2 domain of STAT3. After docking, JAK activity induces the tyrosine phosphorylation of STAT3. The phosphorylated STAT3 proteins finally result in a series of changes of cell biology.

Receptor tyrosine kinases (RTKs) can catalyze the phosphorylation of STAT3 via its intrinsic tyrosine kinase activity in the receptor. The more common receptors include EGFR, VEGFR, PDGFR, and colony stimulating factor-1. Similar to RTKs, non-receptor tyrosine kinases (nRTKs) can also directly phosphorylate STAT3 through transferring a phosphate group from ATP to the tyrosine residue of STAT3. The well-known nRTKs include SFKs and ABl. These two types of kinase both can induce STAT3 to undergo activation, dimerization, transportation to the nuclear and then regulate the corresponding target genes.

### G-protein-coupled receptors/ Rho GTPase family /cadherin engagement

GPCRs are the largest family of membrane proteins that mediate in the signal transduction from the extracellular to intracellular space. GPCRs transmit signals not only via secondary messengers but also transcription factors. Recently, JAKs and STATs have been identified as novel downstream effectors of different heterotrimeric G proteins. Several GPCRs, such as angiotensin II (Ang II)^4^ and S1PR1/2^5^, mediate STAT3 activation by JAKs.

Recent studies suggest that Rac1 which belongs to the Rho GTPase family has an essential role in STAT3 tyrosine phosphorylation. As an effector of Rac1, the evolutionarily conserved male germ cell RacGAP (MgcRacGAP) binds to the DNA-binding domain of STAT3 via its cysteine-rich and GAP domains; the MgcRacGAP–STAT3 association with the IL-6R/gp130 complex mediates the phosphorylation of STAT3 induced by IL-6^6^,^7^. In addition to tyr705 phosphorylation, the Rac1/MgcRacGAP complex may also be involved in STAT3 translocation to the nucleus.

Recently, cadherin engagement has been revealed as a new pathway to activate STAT3. Work from several laboratories indicated that the cell density can cause a sharp increase in STAT3 phosphorylation in breast carcinoma, head and neck squamous cell carcinoma, and normal epithelial cells. After cadherin engagement, Rac1/Cdc42 (another member of the Rho GTPase family) is dramatically activated, and then trans-activates NF-κB and increases the expression of IL6, which is responsible for the observed STAT3 activation^8^,^9^.

### Toll-like receptors

STAT3 is also directly activated by TLR stimulation during the production of IgG by human B cell^10^ and TLR mediated STAT3 activation is required for antibody production and IL-10 production^10^. As a classical activator of TLR4, the lipopolysaccharide (LPS) can remarkably increase the level of phosphorylated STAT3 in the human bladder cancer T24 cell line, suggesting the activation of STAT3 by TLR4 signaling^11^.

The activation of TLR3 during oxidative stress protects photoreceptor survival and visual function. In this TLR3 protection during injury, STAT3 is activated and has a critical role^12^,^13^. STAT3 activation is also correlated with the high expression of TLR2 in tumor tissue^14^. In addition, TLR7 ligation can also induce STAT3 activation and interface with Notch, as well as the canonical NF-κB and MAP kinase pathways^15^.

In addition to cytokines and growth factors, CpG can directly activate STAT3 within minutes via TLR9. This finding reveals a second mechanism by which STAT3 mediates immunosuppression^16^ while creating a potent checkpoint or inhibitor of antitumor immune response^17^. The mechanism by which TLR9 activates STAT3 was recently demonstrated. JAK2 is recruited by Frizzled 4 (FZD4) and then is activated depending on the TLR9 engagement with CpG oligodeoxynucleotidesve (ODNs) and this links CpG–TLR9–FZD4 signaling with subsequent STAT3 tyrosine phosphorylation^18^.

### miRNAs

Recent studies have indicated that miRNAs are critical regulators of STAT3 signaling in the pathogenesis of cancer. MiR-519d functions as a tumor suppressor in breast cancer by suppressing STAT3 expression^19^. The low expression level of miR-20a, a negative regulator of STAT3, can enhance de-repressed STAT3 expression and activation and boost proliferation pathways in hepatocellular and this suggest miR-20a may represent a novel potential therapeutic target and biomarker for survival of cancer patients^20^.

Let-7 miRNA family members are widely considered to be tumor suppressors. Let-7 re-expression in poorly differentiated PDAC cell lines can enhance the cytoplasmic expression of suppressor of cytokine signaling 3 (SOCS3), which blocks STAT3 activation by JAK2, and reduce the phosphorylation of STAT3 and its downstream signaling events, thereby reduce the growth and migration of PDAC cells^21^. Iliopoulos and colleagues revealed that Src activation triggers a nuclear factor (NF)-κB-mediated inflammatory response that directly activates LIN28 transcription, which leads to let-7 inhibition and causes a high expression of IL-6 coupled with the activation of STAT3. Their study demonstrated that the interaction of let-7 and IL-6–STAT3 completes a negative-feedback loop in cellular transformation and first describes the importance of epigenetic regulation in promoting inflammation and cancer^22^. Additionally, downregulation of miR-200 and let-7 via STAT3 can induce the EMT phenomenon in breast cancer; conversely, inactivation of STAT3 or re-expression of both miRNAs proved sufficient to induce mesenchymal-to-epithelial transition (MET) in mesenchymal breast cancer^23^.

### Tyrosine phosphatases

Tyrosine phosphorylation catalyzed by tyrosine kinases (PTKs) is critical for STAT3 activation. By contrast, the dephosphorylation of STAT3 by PTPs including SHP2, SHP1, CD45, PTP1B, PTP2B and PTPRT, are essential to ensure proper amplitudes and kinetics of STAT3 activation^24^. SHP2 negatively regulating STAT3 was observed in melanoma cells and glioma cells^25^,^26^, and morin inhibits STAT3 tyrosine 705 phosphorylation in tumor cells through activation of protein tyrosine phosphatase SHP1^27^. In addition, adiponectin significantly inhibits leptin-induced JAK2 activation and STAT3 transcriptional activity via increasing PTP1B protein and activity in oesophageal cancer cells^28^.

### PIAS protein family

The protein inhibitor of activated STAT (PIAS) proteins which vary between 507 (PIASy) and 650 (PIAS1) amino acid residues are encoded by four genes, namely, PIAS1, PIASx (PIAS2), PIAS3, and PIASy (PIAS4). PIAS proteins regulate transcription through several mechanisms, including blocking the DNA-binding activityof transcription factors, recruiting transcriptional co-repressors, and promoting protein SUMOylation. Recent studies showed that PIAS proteins can deregulate the activity of STAT3^29^,^30^,^31^.

### SOCS protein family

SOCS (Suppressor of cytokine signaling) contain eight members (CIS, SOCS1, SOCS2, SOCS3, SOCS4, SOCS5, SOCS6, and SOCS7^32^). The SOCS proteins negatively regulate the JAK-STAT3 signaling pathway through three mechanisms: first, by inhibiting JAK kinase or target JAKs for degradation by proteasome; second, by shielding the STAT3 binding sites on the cytokine receptor; third, by targeting proteins for proteasomal degradation via ubiquitination. Among those proteins, SOCS1 and SOCS3 are the best characterized so far. A recent study showed that SOCS1 and SOCS3 can promote myogenic differentiation by inhibiting JAK1 and gp130, respectively^30^. Platelet factor 4 (PF4) inhibits the IL-17/STAT3 pathway by upregulating the expression of SOCS3^33^.

### Other regulation patterns of STAT3

**Figure 1:**
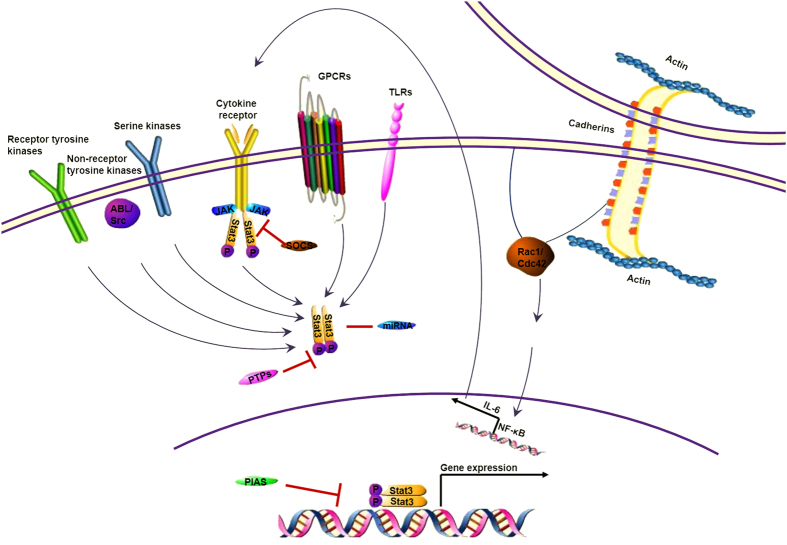
Multiple regulation pathways of STAT3 in cancer. Cytokine receptors, especially receptors for IL-6 family cytokines, are the most well-known traditional activators of STAT3. Receptor tyrosine kinases, non-Receptor tyrosine kinases and some serine kinases can also regulate the STAT3 activity. Recently, studies found that GPCRs and TLRs are involved in the regulation of STAT3. Cadherin engagement accompanied with the high level of Rac1/Cdc42 also dramatically regulates STAT3 through activating the NF-κB signal pathway. SOCS inhibits STAT3 signaling via blockade of upstream signaling through interactions with gp130 and JAK family members. Various miRNAs either restrict or enhance STAT3 signaling. PTPases dephosphorylate STAT3 and prevents dimer formation. PIAS proteins directly compete with STAT3 for either binding opportunities with the activating receptor or for dimerization and translocation into the nucleus.

Apart from the phosphorylation at Tyr705, STAT3 can also be activated by the phosphorylation of Ser727. Various serine kinases, such as MAPK (p38MAPK, ERK, and JNK), PKCδ, mTOR, NLK, and an H-7–sensitive kinase, have been reported to phosphorylate STAT3 at the serine 727, which is required for STAT3 maximal transcriptional activity^34^,^35^,^36^,^37^,^38^,^39^. NF-κB activation is a well-known player that promotes the production of IL-6, which stimulates STAT3 activation. Interestingly, a recent study indicated that STAT3 was responsible for eliciting constitutive NF-κB activity in human melanoma and prostate cancer cells^40^. This finding reveals a STAT3 → NF-κB → IL-6 feed-forward signaling loop in carcinogenesis; meanwhile, the molecular mechanism linking inflammation to cancer was gradually clarified^41^,^42^,^43^. Other factors such as UV radiation or sun light, carcinogen, stress, smoke, and infection are also known to have a significant role in STAT3 activation. (Fig. 1.)

## STAT3 regulates gene expression through epigenetic modification during cancer progression

Although the persistent phosphorylated form of STAT3 has been found in several cancers and leads to gene expression promoting cell proliferation and resistance to apoptosis, as well as tumor angiogenesis, invasion, and migration, unphosphorylated STAT3 also acts as a weak but potentially biologically relevant transcription factor that can activate a series of STAT3 target genes^44^,^45^ through direct binding to a responsive GAS promoter and promotes the development of cancer. The mechanism of unphosphorylated STAT3 target DNA binding is by regulating chromatin organization and binding to AT-rich DNA sequences, which play important roles in regulation of gene expression and/or chromatin organization because of their special structure with a narrow minor groove that can be recognized by proteins^46^. Unphosphorylated STAT92E proteins can maintain heterochromatin via the regulation of histone H3 Lys-9trimethylation (H3K9me3) in Drosophila^47^,^48^. The epigenetic modification function of unphosphorylated STAT3 was also identified by Timofeeva^1^ who suggested that cancer cells such as DU145 and MCF-7 cells have a more open or, at least, more accessible chromatin conformation than the non-transformed MCF-10A cells, thereby allowing for unphosphorylated STAT3 binding, suppression of CHOP expression, and subsequent inhibition of the apoptosis of cancer cells with the involvement of the N-terminal domain of STAT3.

The epigenetic gene silencing effect of gene promoter region mediated through the CpG methylation by DNA methyltransferase 1 (DNMT1) and other members of the DNMT family have key roles in the inhibition of tumor-suppressor gene expression in cancer cells. STAT3 acetylation, another activation form of STAT3, can also contribute to regulate DNMT1 binding to several tumor-suppressor gene promoters and promote the promoter methylation of relative genes and the development of cancer. STAT3 is acetylated on a single lysine residue, Lys685, by its co-activator p300/CREB-binding protein (CBP) in response to cytokine treatment, such as IL-6, LIF, and OSM^49^,^50^,^51^. Acetylated STAT3 induces promoter gene methylation of a major tumor-suppressor gene*, ARHI*, and thereby leads to the low expression level of *ARHI* and promotes cancer cell proliferation in ovarian cancer^2^. Several other tumor-suppressor genes, including *CDKN2* cell *A, DLEC1, STAT1*, and *PTPN6*, can also be induced promoter methylation by acetylated STAT3 in cancer cell lines^3^.

## Pivotal biological functions of STAT3 in cancer

### STAT3 is involved in EMT promoting cancer invasion and metastasis

As a phenotypic switch, EMT is characterized by cells losing the epithelial polarity and acquiring mesenchymal characteristic resulting in the decrease of cell-cell junction and promoting the invasion and metastasis ability of cells. Enough evidences show that the EMT phenotypes plays a significant role in promoting progression of many cancers, such as non-small cell lung cancer (NSCLC)^52^, ovarian carcinomas^53^, hepatocellular carcinomas(HCC)^54^, breast cancer^55^, nasopharyngeal cancer^56^. Various studies demonstrated that STAT3 can modulate the expression of EMT-related transcription factors (Twist, Snail, ZEB1, etc.) and thereby influence the EMT phenotypes. For instance, in HCC cells, STAT3 was revealed to bind to the promoter of Twist, mediate its transcriptional activity, and then promote the EMT process and increase the cells invasion and migration ability for the first time^57^. In bresat cancer, STAT3 activation by EGF treatment induced higher Snail expression and the high expression level of snail was reversed by N-myc downstream-regulated gene 2 (NDRG2) through inhibits STAT3 binding to the Snail promoter and subsequently inhibit the EMT process and cancer progression^58^. Additionally, prolonged activation of STAT3 leads to low expression of let-7 and miR-200 coupled with the upregulation of ZEB1 in OSM-triggered EMT, which contributes to the acquisition of the mesenchymal phenotype and invasive capability as well as promotion of breast cancer progression^23^. These findings suggest that STAT3 responds to the integrating signals from multiple extracellular stimuli that influence the EMT phenotype, regulates the level of EMT-related transcription factors, and enhances the cancer cell abilities of invasion, metastasis. Therefore, targeting STAT3 may provide a means to reverse the EMT phenotypes and prevent cancer invasion and metastasis.

### STAT3 in tumor microenvironment

As a major regulator of tumorigenesis, tyrosine phosphorylated STAT3 has been detected and is mainly distributed on the leading edge of tumors in association with stromal, immune, and endothelial cells^59^. This result effectively suggests that STAT3 has a critical role in the communication between cancer cells and their microenvironment which has the following aspects. (A) Production of humoral factors. For instance, the paracrine sources of IL-6 from cancer-associated fibroblasts, adipocytes, or myeloid cells on the edge of the tumors and the autocrine production of IL-6 can both activate the STAT3. The pSTAT3 in turn promotes the expression of IL-6, thus forming amplification loops of the production of IL-6, which can induce the vast expression of autocrine and paracrine cytokines and growth factors, including IL-8, CCL5, CCL2, CCL3, IL1-β, GM-CSF, VEGF, and MCP-1, which are highly expressed and play an important role in the generation and development of cancer. (B) Interaction with fibroblasts, adipocytes and macrophages. Cancer-associated fibroblasts (CAFs) can promote cancer progression via remodeling the ECM, induction of angiogenesis, recruitment of inflammatory cells, and directly stimulating cancer cell proliferation via the secretion of growth factors and mesenchymal–epithelial cell interactions, which are mainly regulated by the IL-6–STAT3–Twist signaling pathway by upregulating the expression of CXCL12^60^, a Twist target gene associated with the regulation of the CAF phenotype. When cancer-associated adipocytes separated from the breast cancer patients co-culture with MCF7 and MDA-MB-231 breast cancer cell lines, adipocytes revert to an immature and proliferative phenotype, and promote cancer cell migration via the high expression of IL-6^61^. Due to the protumoral functions, tumor-associated macrophages have received much attention as novel cancer target cells. Researchers shown that suppressing STAT3 activation by triterpenoid compounds can inhibited macrophage polarization to M2 phenotype which are involved in tumor development and poor clinical prognosis^62^. (C) Promotion of immune suppression. Cancer cells regulate their immunological environment, recruit immune cells, subvert their functions to their own advantage, prevent them from mounting an effective immune response and instead promote cancer progression. STAT3 is an established molecular hub of immune suppression. STAT3-regulated genes encoding VEGF and IL-10 mediate crosstalk between cancer cells and contribute to a state of immunosuppression^63^. STAT3 promotes myeloid-derived suppressor cells (MDSCs) expansion and immune suppression in lung cancer^64^ and exosomal Hsp70 mediates immune suppression activity of MDSCs via p-STAT3^65^. Blocking the STAT3 activity is in favor of reversing the hepatocellular carcinoma-induced immune suppression and enhancing the NK cell functions^66^. (D) Linking inflammation to cancer. STAT3 and NF-κB are two important transcription factors; both function as critical regulators in inflammation and cancer development, with vital roles to control the communication between cancer cells and inflammatory cells. They cooperatively bind at a subset of gene promoters and synergistically induce their target gene expression and function in the process of inflammation and tumorigenesis. Pro-inflammation cytokines induced by NF-κB or STAT3 can positively feedback to induce STAT3 and NF-κB activation^42^ and promote cancer progression. The collaborative functions of STAT3 and NF-κB make a link from inflammation to cancer and the mechanism inflammatory responses have a decisive role at different stages of tumor development becomes gradually clear. This may provide a new treatment strategy for cancer. (E) Tumor angiogenesis. Growing evidence indicates that activated STAT3 participates in angiogenesis regulation, with a critical role^67^. VEGF and bFGF are known to be involved in endothelial cell proliferation, extracellular matrix degradation, endothelial cell migration, and modulation of junctional adhesion molecules; both have been described as the leading mediators of angiogenesis that can be upregulated by activated STAT3 in glioblastoma stem cells^68^, papillary thyroid cancer^69^, and colorectal cancer^70^, thereby promoting the formation of new blood vessels and development of cancer. STAT3 is involved in various aspects of tumor microenvironment to cultivate a favorable environment for cancer development. Targeting STAT3 present a feasible stategy to weaken the supporting function of tumor microenvironment and improving the therapeutic effect for cancer.

### STAT3 regulates CSCs

Given its important role in sustaining the self-renewal and differentiation of Embryonic Stem CellS (ESCs)^71^,^72^,^73^, STAT3 is also evidently essential for regulating CSCs of cancers such as ovarian cancer^74^, HCC^75^, breast cancer^76^ colorectal cancer^77^, glioblastoma^78^, lung cancer^79^, and prostate cancer^80^. The STAT3 regulatory mechanism of stem cell self-renewal and differentiation is mainly focused on the ESC-specific roles of LIF. When LIF binds to LIFR and gp130, the heterodimerized compound can activate STAT3 and then, birdged by Bcl3 to the Oct4 signaling and maintain pluripotency of ESCs^71^,^81^. Other IL-6 family members, such as OSM, CNTF, CTF-1, and CLC, which form heterodimerization of gp130 with the LIF receptor, can also maintain the self-renewal and differentiation of stem cells because of their shared signaling mechanisms that converge on STAT3.

As a multifunctional cytokine, IL-6 has been implicated in the maintenance of stem cancer cells through the IL-6/gp130/STAT3 signaling pathway. In gene expression profiles of CD44^+^/CD24^–^ breast CSCs, IL-6 has been demonstrated to be upregulated^15^. Liu^79^ showed that IL-6/JAK2/STAT3 pathway upregulates DNMT1 and enhances cancer initiation and lung CSC proliferation via the downregulation of p53 and p21, which results from DNA hypermethylation. In addition, IL-6/STAT3/NF-κB signaling pathways are both activated in CSCs and its microenvironment^82^,^83^. Activation of these pathways stimulates further cytokine production and generates positive feedback loops that in turn drive CSC self-renewal. Furthermore, the constitutive activation of STAT3/NF-κB signaling can regulate the Notch pathway, which appears to play a key role in CSCs in a variety of cancers and controls cell fate determination, survival, proliferation, and the maintenance of stem cells^84^.

Although the activation of STAT3 via IL-6 has been identified as necessary for promoting CSC-like phenotypes, other STAT3 activators are also involved in the regulation of CSCs. Conti^85^ first showed the role of TLR2 in mammary CSC self-renewal through binding to its receptor HMGB1, increasing the secretion of IL-6 and subsequently activating the STAT3 signaling pathway. Downregulation of miR-1181 can promote CSC-like phenotypes in human pancreatic cancer by promoting the STAT3 signaling pathway and the activation of the CSC transcription factor SOX2^86^. RhoC expression is found to be correlated to CSC formation in head and neck squamous cell carcinoma (HNSCC). RhoC elevates the expression level of IL-6 and then promotes the phosphorylation of STAT3^ser727^and STAT3^tyr705^ as well as the high expression of Nanog, oct3/4, and sox2 in HNSCC^87^. In addition, a novel EGFR/STAT3/Sox-2 paracrine signaling pathway which is required for macrophage-induced upregulation of Sox-2 and CSC phenotypes in tumor cells is identified^88^.

### STAT3 inhibitors

STAT3 is considered as an ideal molecular target of cancer therapy because this target plays a pivotal role in tumorigenesis and cancer cell biology. As such, great efforts have been devoted to the discovery of potent and selective inhibitors that target STAT3. STAT3 inhibitors are divided into two types depending on whether the activity of STAT3 can be inhibited indirectly or directly. Indirect inhibitors block upstream effectors, such as cytokine and kinases, involved in STAT3 activation. For instance, ALD518, a humanized anti-IL-6 antibody, helps NSCLC patients obtain therapeutic benefits against cachexia, anemia, and drug resistance^89^. WP1066, a JAK2 inhibitor, suppresses ovarian cancer growth, migration, and invasion; this inhibitor also enhances the chemosensitivity of ovarian cancer cells and decreases the rate of STAT3 phosphorylation^90^. Direct inhibitors directly block the SH2, DNA-binding, and N-terminal domains of STAT3 to suppress protein dimerization, to inhibit DNA binding, and to prevent nuclear translocation, respectively. Among these domains, the SH2 domain has been considered as the most commonly investigated site because of its critical involvement in STAT3 activation; furthermore, inhibitors targeting the SH2 domain constitute the largest class of direct inhibitors.

**Figure 2:**
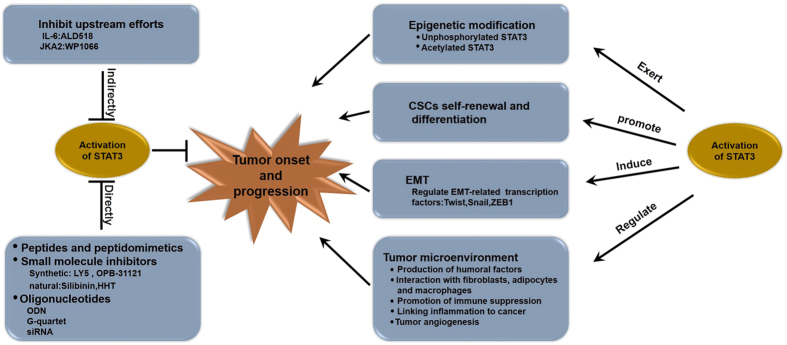
Pivotal biologiccal functions in cancer and inhibitors of STAT3. STAT3 plays a pivotal role in tumor onset and progression through altering gene expression via epigenetic modification, inducing EMT phenotypes in cancer cells, regulating the tumor microenvironment, and promoting CSCs self-renewal and differentiation. As an ideal target for cancer therapy, lots of indirect or direct inhibitors for STAT3 have been developed recently.

Inhibitors are also divided into three classes of compounds on the basis of structure. (A) One of these classes includes peptides and peptidomimetics. Although peptides and peptidomimetics can directly disrupt the dimerization of STAT3 and effectively inhibit its transcriptional activity, these inhibitors present several challenges related to low cell permeability and stability. (B) Another class of these compounds comprises small molecule inhibitors. With advances in medicinal chemistry and structural applications based on high-throughput virtual screening and site-directed computational fragment-based drug design approach in silico, small-molecule STAT3 inhibitors, which overcome problems related to cell permeability, show much feasibility to inhibit the STAT3 activity. Novel synthetic or natural small-molecule STAT3 inhibitors have been evaluated by using preclinical models. LY5, a novel non-peptide, cell-permeable small molecule inhibitor of STAT3 dimerization, blocks STAT3 activation with low IC50 values (0.5–1.4 M) and strong binding affinity to the STAT3 SH2 domain. LY5 selectively inhibits persistent STAT3 activation and induces the apoptosis of medulloblastoma cells and becomes a promising therapeutic drug candidate for human medulloblastoma by inhibiting STAT3 signaling^91^. OPB-31121, an inhibitor assessed through active clinical trials, interacts and exhibits a high affinity to the SH2 domain of STAT3^92^ and elicits a significant antitumor effect on leukemia^93^ and gastric cancer^94^. Silibinin, a natural polyphenolic flavonoid extracted from the seeds of milk thistle (Silybum marianum), is an optimum inhibitor of pSTAT3 in gastric^95^, breast cancer^96^, prostate^97^ in preclinical studies and clinical trials related to this flavonoid have also been conducted. However, therapeutic effects on cancer patients remain unsatisfactory because the bioavailability of its flavonolignan structure is low^98^. Homoharringtonine (HHT), another natural compound extracted from Cephalotaxus harringtonia, significantly inhibits the STAT3 activity by suppressing the IL-6/JAK1/STAT3 signaling pathway and induces the apoptosis of Gefiinib-resistant lung cancer cells. *In vivo*, HHT remarkably suppresses the tumor growth but Gefiinib does not exhibit comparative effect in nude mice injected H1975 cells and this identifies the HTT as a novel potential natural inhibitors for patients with NSCLC in a EGFR-independent manner^99^. (C) Oligonucleotides. Because of the application of advanced molecular techniques, oligonucleotides inhibitors targeting STAT3 seem viable to selectively inhibit STAT3 activity. Decoy Oligonucleotide (ODN) usually is a double stranded 10–20 base pair DNA containing a TF’s consensus and selectively inhibits STAT3 activity by competitively binding to the DNA binding domain of STAT3; thus, specific gene expression is effectively attenuated. First-in-human trial of a STAT3 decoy oligonucleotide in head and neck tumors has been recently assessed. With improvements in cyclization, STAT3-targeting ODN seems more amenable to systemic administration and can yield optimum effects by downregulating STAT3 target genes and by suppressing tumor growth^100^. G-quartet oligonucleotides are G-rich oligodeoxynucleotides that form four-stranded potassium-dependent intramolecular G-quartet structures and occupy sites within the SH2 domains of STAT3. These oligonucleotides effectively inhibit Stat3 activation and tumor growth in head and neck cancer^101^, NSCLC^102^, Prostate Cancer^103^. Nonetheless, their large size and potassium dependence limit their cellular delivery and possibility to be assessed in clinical trials. Small interfering RNA (siRNA) is a natural post-transcriptional gene-silencing mechanism to turn off unwanted genes. Targeting STAT3 using siRNA represent a useful approach for the treatment of breast cancer^104^, lung adenocarcinoma^105^. However, further studies should be conducted regarding STAT3 silencing for cancer therapy. (Fig. 2.)

### Conclusion and future directions

Although, STAT3 is an ideal target of cancer therapy because of its multiple regulatory pathways and pivotal biological functions in cancer; furthermore, various inhibitors targeting STAT3 have been developed for cancer therapy, no candidate compounds are potent enough to provide beneficial therapeutic effects for cancer patients. As such, new directions for cancer therapy by targeting STAT3 should be explored. For instance, small-molecule inhibitors of GPCR, TLR, and miRNAs related to STAT3 regulation can be applied to treat cancer. Current anticancer-targeted therapeutics mainly focuses on inhibiting the tyrosine phosphorylation of STAT3, however, epigenetic modification function of STAT3 may present a novel and powerful therapeutic approach for cancer treatment. Therefore, further studies should be conducted to address the questions regarding STAT3 in cancer and to find the best efficient strategies that can inhibit the STAT3 activity to gain optimum therapeutic effects.

## Additional Information

**How to cite this article**: Yuan, J. *et al*. Multiple regulation pathways and pivotal biological functions of STAT3 in cancer. *Sci. Rep*. **5**, 17663; doi: 10.1038/srep17663 (2015).
